# Dr. Washington H. Baker

**Published:** 1904-05

**Authors:** 


Dr. Washington H. Baker, of Philadelphia, died at his home in that city, April 1, 1904, aged 52 years. He graduated at the University of Pennsylvania in 1875. He was a fellow of the American Association of Obstetricians and Gynecologists.

